# Effect of Yoga Practices on Postural Stability, Fall Risk, and Psychological Wellbeing in Older Adults

**DOI:** 10.3390/geriatrics11010016

**Published:** 2026-02-05

**Authors:** Sanjay Shete, Anita Verma, Ranjeet Singh Bhogal, Subodh Tiwari

**Affiliations:** Kaivalyadhma Yoga Institute, Swami Kuvalayananda Marg, Lonavla 410403, Pune, India; sanjays@kdham.com (S.S.); bhogal@kdham.com (R.S.B.); subodh@kdham.com (S.T.)

**Keywords:** yoga, older adults, balance, falls, functional mobility, depression, anxiety, mental health

## Abstract

**Background:** Advancing age is frequently associated with balance impairment, increased fall risk, and psychological distress, which together contribute to loss of independence and reduced quality of life. Yoga, as a mind–body practice, has the potential to enhance physical stability as well as mental well-being in older adults. Therefore, the objective of this study was to evaluate the effects of a structured yoga program on balance, fear of falling, mobility, and mental health outcomes among older adults. **Methods:** A quasi-experimental pretest–post-test study was conducted at Nagpur, India. A total of 64 eligible participants (65–85 years) were purposively assigned to a yoga intervention group (n = 32) or a waitlist control group (n = 32). The 12-week intervention comprised preparatory exercises, yoga postures, breathing practices, and meditation. Outcomes assessed at baseline and post-intervention included balance, fear of falling, mobility, depression, and anxiety. **Results:** Data from 50 participants (yoga: n = 26; control: n = 24) were analyzed. The yoga group showed significant improvements in balance (*p* < 0.001) and functional mobility (*p* < 0.001), with significant reductions in fear of falling (*p* = 0.009), anxiety (*p* = 0.0003), and depression (*p* = 0.004). In contrast, the control group exhibited deterioration in functional mobility (*p* = 0.001) and anxiety (*p* = 0.009), with no significant gains in other measures. Between-group comparisons confirmed significantly greater improvements in the yoga group across all outcomes. **Conclusions:** A 12-week yoga program was feasible and effective in improving balance, functional mobility, and mental health, while reducing fear of falling among older adults. Yoga may serve as a safe, non-pharmacological intervention to promote healthy aging in institutionalized populations. **Trial registration:** This study was prospectively registered with the Clinical Trial Registry of India (Registration No: CTRI/2023/10/058682; Registered on: 16 October 2023).

## 1. Introduction

As persons grow older, the goals of maintaining functional abilities, social independence, and cognitive abilities become increasingly challenging and important. Aging is often accompanied by multiple medical conditions, which can further contribute to declines in both physical and mental health among older adults [1,2]. World Health Organization defines active aging as one important factor to optimize health and to enhance quality of life, and it promotes physically active lifestyles in older adults [3]. Factors affecting physical and mental health in geriatric population of India are socioeconomic status [4,5], nucleation of family system [6], gender [7,8], illiteracy, marital status [9], malnutrition [10], urban environment, family support, and physical inactivity [11,12]. Older adults commonly experience conditions, such as osteoarthritis, chronic obstructive airway disease, anemia, hypertension, cataract, dental problems, hearing loss, gastrointestinal disorders, genitourinary disorders, neurological disorders [9,13], depression, anxiety, memory loss, dementia, and bipolar disorder [8].

Additionally, gait disturbance is a common medical problem in old age [14]. Problems of balance and gait are associated with immobility and falls, which markedly impair the quality of life. Deterioration in balance function, whether due to disease or aging, increases the risk of balance loss and falls [15]. Since gait and balance directly influence an individual’s capacity to remain mobile and safe, they are closely linked with functional ability in older adults. Previous studies have demonstrated significant associations among functional ability, slow gait initiation time, and falling [16,17]. Therefore, functional ability represents a more significant and holistic construct than merely assessing balance or walking speed alone.

Functional ability is the ability to perform basic activities of daily life without support, which is the key to overall independence and quality of life. Impairment in functional ability reflects a reduced capacity to meet daily needs without assistance [18]. Such impairments may affect the quality of life considerably and influence future care of the geriatric population [19]. Maintaining functional ability is therefore a critical indicator of health status in older adults, whose independence is often easily compromised by illness and age-related decline.

Perceiving this problem in older adults, yoga may be introduced as a training intervention, as it has been shown to positively influence functional abilities through its effects on balance, stretching, strengthening, and relaxation [20,21]. Hence, this study was planned to examine the effect of yoga practices on balance, fear of falls, and mental health in the geriatric population, while also encouraging older adults to adopt lifestyle modification programs. Previous research studies indicate that a physically active lifestyle is associated with improvements in fall risk factors [22,23,24], and functional ability [25,26,27,28], as well as gait and balance [29,30,31,32,33,34]. Regular physical activity has also been shown to enhance brain function, neuroplasticity, and mental health outcomes, including reductions in depression and anxiety, among older adults [35,36,37].

Earlier research studies indicate that falls among elderly individuals are influenced by multiple factors. Importantly, fear of falling and impaired balance are potentially modifiable with appropriate approaches such as yoga and other physical activities. However, despite growing interest in mind–body interventions, limited research has specifically examined the effects of yoga practices in institutionalized geriatric populations. Therefore, the present study was conducted with the objective to evaluate the effect of yoga on balance, risk of falls, functional mobility, and mental health in older adults. Based on existing evidence, it was hypothesized that older adults who participated in the structured yoga intervention would demonstrate significantly greater improvements in balance, functional mobility, fear of falling, and mental health outcomes compared with those in the waitlist control group.

## 2. Materials and Methods

### 2.1. Study Design and Setting

The study employed a quasi-experimental pretest–post-test design with an experimental (yoga) group and a waitlist control group. Participants were recruited purposively from a single site, Soham Old Age Home, a residential care facility in Nagpur, Maharashtra, India. The study was conducted between November 2024 and February 2025. The study protocol was approved by the Institutional Ethical Committee of Kaivalyadhama, Lonavala (Reference No: Kdham/SRD/IEC-04/2023/01) and was prospectively registered with the Clinical Trial Registry of India (Registration No: CTRI/2023/10/058682; Registered on: 16 October 2023). All procedures performed were in accordance with the ethical standards of the institutional committee and with the 1964 Helsinki declaration.

### 2.2. Participants

A total of 69 older adults from the participating old age home was assessed for eligibility. The sample size was based on feasibility within the residential care setting and on participant availability during the study period, rather than on a formal a priori power calculation. Individuals were included if they were (1) aged between 65 and 85 years, (2) able to walk (with or without a walking aid), and (3) provided written informed consent. Exclusion criteria included (1) significant mobility issues that would make yoga practice unsafe, (2) any known serious or unstable health condition (e.g., unstable cardiovascular disease, severe osteoporosis) as assessed by a medical practitioner, or (3) current regular yoga practice.

Out of 69 older adults, 64 eligible participants were enrolled in the study. They were allocated to either the yoga intervention group (n = 32) or the waitlist control group (n = 32) through purposive sampling and convenience-based group assignment. Group allocation was based on participants’ availability and willingness to attend daily morning yoga sessions, as well as logistical feasibility within the residential care setting. Although this non-random allocation may introduce selection bias, baseline demographic and clinical characteristics were largely comparable between groups. Additionally, analysis of covariance (ANCOVA) was used to adjust for baseline outcome values to reduce the potential influence of pre-existing group difference.

### 2.3. Assessment

Data on balance, fall, depression, anxiety and functional mobility were collected at baseline and immediately after the 3-month yoga intervention period using validated tools.

Berg Balance Scale (BBS): Balance was assessed using the BBS, a 14-item objective measure that evaluates static and dynamic balance abilities through common tasks of daily living. Each item is scored on an ordinal scale from 0 (cannot perform) to 4 (normal performance), with a total maximum score of 56. Higher scores indicate better balance performance. The BBS has been demonstrated to be a valid and reliable tool for assessing balance in older adults [38].

Fall Efficacy Scale (FES): The fear of falling was evaluated using the Fall Efficacy Scale. This self-report questionnaire assesses an individual’s level of concern about falling during 16 common daily activities. Each activity is rated on a 4-point Likert scale ranging from 1 (not at all concerned) to 4 (very concerned). The total score ranges from 16 to 64, with higher scores indicating a greater fear of falling. The FES has been found to be a psychometrically sound instrument for use among older adults [39].

Geriatric Depression Scale (GDS-15): Depressive symptoms were screened using the 15-item Geriatric Depression Scale. This is a yes/no self-report measure where participants indicate if they have felt a certain way over the past week. Scores range from 0 to 15, with higher scores suggesting more severe depressive symptoms. The short form GDS is widely used and has been validated for the assessment of depression in older adults [40].

Geriatric Anxiety Scale—10 Item Version (GAS-10): Anxiety symptoms were measured using the 10-item Geriatric Anxiety Scale (GAS-10), a shortened version derived from the original 30-item scale. This tool is specifically designed to assess common anxiety symptoms in older populations. Respondents rate how often they have experienced each symptom over the past week (e.g., “felt nervous”, “worried too much”) on a 4-point Likert scale ranging from 0 (Not at all) to 3 (All of the time). The total score ranges from 0 to 30, with higher scores indicating greater levels of anxiety. The GAS-10 has been validated for its psychometric properties and is found to be a reliable and efficient screening tool for anxiety in older adults [41].

Timed Up and Go Test (TUG): Functional mobility was assessed with the Timed Up and Go Test. Participants were instructed to stand up from a standard armchair, walk a distance of 3 m at their usual pace, turn around, walk back to the chair, and sit down again. The time taken to complete this sequence was measured in seconds using a stopwatch. Longer completion times indicate poorer mobility and higher risk of falls. The TUG is a well-established, reliable, and valid measure for older adults [42].

### 2.4. Data Collection Procedure

To facilitate data collection, ten postgraduate students from Nagpur, preparing for doctoral studies were recruited and given comprehensive training by the study researchers. A dedicated training session was conducted for two weeks to instruct these students in efficient test administration, including a demonstration of proper techniques. The students were instructed to thoroughly practice administration procedures during training, and subsequently, the same trained individuals conducted test administration at both baseline and post-intervention time points under the supervision of research team.

### 2.5. Intervention

The yoga intervention was administered over a total of 12 weeks and was structured into two distinct phases to ensure safety, adherence, and gradual progression for older adults. The training sessions were conducted daily in the morning from 6:30 am to 7:30 am.

Phase 1: Preparatory Training (Weeks 1–4): Considering the advanced average age of the participants (approx. 75 years) and the prevalent initial physical limitations such as stiffness and reduced joint mobility, the first month was designated as a preparatory phase. The primary objective of this phase was to gently enhance joint flexibility, improve circulation, and build strength, thereby preparing the participants’ bodies for the administration of formal yoga protocol.

The preparatory training phase consisted of a series of *Sukshma vyayam* (warm up exercises) designed to gently enhance joint flexibility and build strength. These exercises were performed primarily in a seated chair position or supported standing to maximize their safety and convenience. This included controlled joint mobilization exercises targeting the knees, shoulders, hips, ankles, and neck through their full comfortable range of motion. Further, these exercises were specifically designed to be complementary to subsequent yoga postures (*asanas*) by systematically addressing stiffness in the joints and major muscle groups.

Phase 2: Structured Yoga Program (Weeks 5–12) Following the preparatory phase, a structured yoga program was implemented for the remaining eight weeks. The protocol was designed to improve balance, strength, flexibility, and mental well-being, and included the following components, performed with appropriate modifications as needed:

1. Asanas (Postures): The sequence included Crocodile variations (*Makarasana*), Half-Plow Pose (*Ardha*-*Halasana*), Half-Wind Relieving Pose (*Ardha*-*Pawanmuktasana*), Bridge Pose (*Setubandhasana*), Unsupported Bridge Pose (*Niralambasana*), Locust Pose (*Shalabhasana*), Cat Pose (*Marjariasana*), Side-Bending Pose (*Vakrasana*), Tree Pose (*Tadasana*), Triangle Pose (*Konasana*), and a final relaxation in Corpse Pose (*Shavasana*).

2. Pranayama (Breathing Practices): Gentle breathing techniques were incorporated, including Alternate Nostril Breathing (*Anulom*-*Vilom*), Skull Shining Breath (*Kapalbhati*), Victorious Breath (*Ujjayi*), and Humming Bee Breath (*Bhramari*).

3. Meditation: Each session concluded with 15 min of guided meditation to promote mental calmness and relaxation.

Participant safety and comfort were paramount throughout the intervention, with several specific measures implemented to prevent fatigue and ensure a gentle practice. These included instructing participants to rest for one minute after each asana and, most crucially, emphasizing that they should not be forced to achieve the final expression of any posture. Instead, all practices were adapted and performed strictly according to each individual’s personal comfort level and physical capability. This participant-centric approach aimed to provide a positive and sustainable practice without risk of strain or injury. Meanwhile, participants allocated to the waitlist control group were instructed to maintain their usual daily activities during the 12-week intervention period and did not receive any special training or structured program.

### 2.6. Statistical Analysis

The normality of the data for all continuous outcome variables (balance, fall risk, anxiety, depression and functional mobility scores) was assessed using the Shapiro–Wilk test. The initial results indicated a significant deviation from normality (*p* < 0.05). However, following the guidance of several statistical authorities, we examined the skewness and kurtosis of the data. For all variables, the absolute values of kurtosis were well below the critical threshold of 3.0, and skewness values were below 2.0, indicating that the distributions were sufficiently normal to proceed with parametric analyses [43,44,45]. Descriptive statistics are presented as mean (±standard deviation) for continuous variables. Baseline demographic and clinical characteristics between the yoga intervention and control groups were compared using independent samples *t*-tests for continuous data and chi-square tests for categorical data.

The primary analysis evaluated the impact of the yoga intervention. Paired samples *t*-tests were used to analyze within-group changes from pre- to post-intervention for each outcome. Between-group differences in post-intervention outcomes were evaluated using analysis of covariance (ANCOVA), with the baseline score for each outcome entered as a covariate to control for initial differences between groups. Effect sizes for within-group changes were expressed as Cohen’s d, and effect sizes for the ANCOVA were reported as partial eta squared. All statistical analyses were performed using SPSS (Version 25.0), with statistical significance set at *p* < 0.05.

## 3. Results

### 3.1. Participant Flow and Attrition

**Figure 1:**
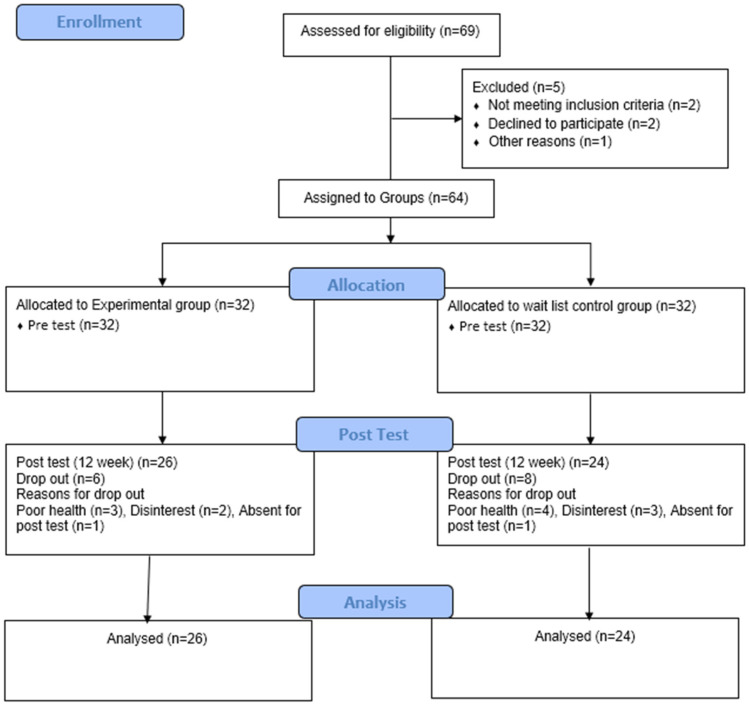
Flow chart of study participants.

A total of 69 older adults expressed willingness to participate in the study. After screening for inclusion and exclusion criteria, 64 individuals were enrolled and allocated to either the yoga intervention group (n = 32) or the waitlist control group (n = 32). During the study period, 14 participants dropped out: 6 from the intervention group and 8 from the control group. Reasons for dropout included illness (n = 4), loss of interest (n = 3), absence during pre- or post-testing (n = 4), and irregular attendance in yoga sessions (n = 3). Consequently, data from 50 participants (intervention: n = 26; control: n = 24) were included in the final analysis (Figure 1).

### 3.2. Baseline Demographic Characteristics

The demographic characteristics of the participants are presented in Table 1. No significant differences were observed between the yoga and control groups at baseline with respect to age, marital status, education level, living arrangement, financial problems, previous occupation, or duration of stay at the old age home (*p* > 0.05 for all). However, a significant difference in gender distribution between the groups was noted.

### 3.3. Within-Group Changes in Outcome Measures

The pre- and post-intervention outcomes for balance, fear of falling, anxiety, depression, and functional mobility, along with adjusted between-group differences and corresponding 95% confidence intervals, are summarized in Table 2. Within-group analyses indicated that participants in the yoga group demonstrated statistically significant improvements from baseline to 3 months across all outcome measures. Significant improvements were observed in balance (t = 4.5, *p* < 0.001) and functional mobility (t = 4.4, *p* < 0.001), along with significant reductions in fear of falling (t = 2.8, *p* < 0.001), anxiety (t = 4.2, *p* < 0.001), and depression (t = 3.1, *p* < 0.001).

In contrast, the control group showed no significant improvement in balance (t = 0.28, *p* = 0.77) or fear of falling (t = 1.39, *p* = 0.17). The control group exhibited a significant deterioration in anxiety (t = 2.81, *p* = 0.009) and functional mobility (t = 3.7, *p* < 0.001), as well as a trend toward increased depression (t = 2.02, *p* = 0.05).

### 3.4. Between-Group Comparisons

Between-group comparisons using ANCOVA, adjusted for baseline scores, indicated that the yoga group achieved significantly more favorable changes than the control group across all outcome measures. The yoga group demonstrated significantly greater improvements in balance (F(1,48) = 14.06, *p* < 0.001) and functional mobility (F(1,48) = 49.61, *p* < 0.001), as well as significantly larger reductions in fear of falling (F(1,48) = 13.02, *p* < 0.001), anxiety (F(1,48) = 22.82, *p* < 0.001), and depression (F(1,48) = 13.70, *p* < 0.001). These findings indicate moderate to large effect sizes in favor of the yoga intervention.

## 4. Discussion

Consistent with the study hypothesis, the present findings indicate that older adults who participated in the structured 12-week yoga program demonstrated significantly greater improvements in balance, fear of falling, anxiety, depression, and functional mobility compared with the waitlist control group. The present study examined the effects of a structured 12-week yoga program on these outcomes in older adults residing in an old age home, and the results support the hypothesized beneficial role of yoga in improving both physical and psychological health parameters in this population.

It is evident from past studies that balance impairment and functional mobility decline are common geriatric concerns, often leading to falls and loss of independence [46,47]. In the current study, yoga participants showed significant improvements in BBS scores and faster completion of the TUG test, while the control group exhibited decline in functional mobility. These findings are in line with prior studies that demonstrated the effectiveness of yoga and yoga-based exercise programs in enhancing postural stability, strength, and proprioception in older adults [48,49]. The incorporation of standing asanas, such as Tadasana and Konasana, together with breathing practices and relaxation techniques, may have contributed to improved postural control and reduced instability. However, these potential mechanisms are inferred based on prior literature, as proprioceptive and neuromuscular functions were not directly assessed in the present study. Importantly, the magnitude of these improvements suggests clinical relevance. Previous literature indicates that an improvement of approximately 4–7 points on the BBS and a reduction of about 3 s on the TUG represent meaningful functional change in older adults. In the present study, the mean improvements in the yoga group met or exceeded these thresholds, indicating that the changes were not only statistically significant but also functionally meaningful. Furthermore, commonly used clinical cut-offs identify BBS scores below 45 and TUG values above 13.5–14 s as indicative of increased fall risk. Post-intervention scores in the yoga group shifted toward or beyond these thresholds, suggesting a clinically meaningful reduction in fall risk. Such outcomes are clinically meaningful because impaired balance and functional mobility strongly predict falls, hospitalizations, and institutionalization among older adults [50]. The present study also found a significant reduction in fear of falling among participants in the yoga group. Fear of falling has been identified as a powerful psychological determinant of functional decline among older adults [51]. An earlier study showed that introducing yoga postures and pranayama in older adults reduces fear of falling, enhances confidence, and improves functional mobility for performing daily activities more efficiently [52]. Reducing fear of falling may further reinforce mobility gains by encouraging greater engagement in physical activity and daily movement.

Improvements in mental health outcomes further strengthen the value of yoga for older adults. Participants in the yoga group reported significant reductions in depression and anxiety symptoms, whereas the control group showed increased anxiety and a trend toward increased depression. These findings are consistent with meta-analyses demonstrating the efficacy of yoga in reducing psychological distress among older adults [53]. In fact, previous studies suggest that pranayama and meditation practices may promote parasympathetic activation, improve vagal tone, and enhance GABA activity, thereby reducing stress and mood disturbances. However, these physiological mechanisms were not directly measured in the present study and are therefore discussed as plausible explanatory pathways [54]. Moreover, the group-based yoga practices may have enhanced social connectedness, an important factor in alleviating loneliness and psychological vulnerability in institutionalized older adults [55]. This cultural and contextual factors may also have influenced the observed adherence and outcomes. In India, yoga is widely recognized and culturally accepted as a traditional health-promoting practice, which may have enhanced participant familiarity, motivation, and engagement with the intervention. This cultural acceptance may partly explain the favorable adherence and positive outcomes observed in the present study. Therefore, caution is warranted when extrapolating these findings to populations in which yoga is less familiar or culturally embedded, and future research should examine the effectiveness of similar interventions across diverse cultural contexts.

The integrative nature of yoga, combining asanas, pranayama, and meditation, makes it particularly suitable for health promotion in older adults. Unlike conventional exercise, yoga is low-impact, adaptable, and inclusive of individuals with limited mobility or chronic conditions, making it sustainable for long-term practice [56]. Recent reviews have highlighted the broad physical and psychological health benefits of yoga for older adults, while also emphasizing that yoga remains underutilized and under-researched in this population [57]. The present study adds to a growing body of evidence that yoga offers simultaneous physical and psychological benefits, addressing the multidimensional health needs of the older population. Although the present study demonstrated significant improvements following a 12-week yoga intervention, the persistence of these benefits beyond the intervention period cannot be determined due to the absence of long-term follow-up. However, previous studies suggest that continued participation in yoga or similar mind–body practices may help maintain gains in balance, functional mobility, and psychological well-being in older adults. Future studies with extended follow-up are required to confirm the durability of these effects.

The present study has several notable strengths. It employed a structured and supervised yoga intervention tailored to older adults, enhancing safety and adherence within an institutional setting. Multiple validated clinical and psychological outcome measures were used, allowing comprehensive assessment of balance, functional mobility, fear of falling, and mental health. Importantly, the study focused on an institutionalized geriatric population, a group that remains underrepresented in yoga and physical activity research. The observed changes were not only statistically significant but also clinically meaningful, strengthening the practical relevance of the findings. Together, these strengths support the potential role of yoga as a feasible and holistic intervention for promoting physical and psychological well-being in older adults.

## 5. Limitations

Despite the encouraging findings, this study has certain limitations. It was conducted in a single residential care facility, which may restrict the generalizability of results to community-dwelling older adults. Therefore, the findings should be generalized to community-dwelling older adults or individuals with more severe mobility impairments with caution, as such populations may require different levels of supervision or protocol modification. As the sample size was determined based on feasibility rather than a formal power calculation, the relatively high attrition rate of 21.8%, with differential dropout between groups, may have introduced attrition bias and limited the ability to detect smaller effect sizes. As post-intervention outcome data were not available for participants who withdrew, intention-to-treat analysis was not feasible, and a per-protocol approach was therefore adopted. Available baseline information did not indicate clear or systematic differences between participants who completed the study and those who withdrew; however, the possibility of unmeasured factors influencing dropout cannot be entirely excluded.

The intervention was limited to 12 weeks, preventing assessment of the long-term sustainability of benefits. Consequently, it remains unclear whether the observed improvements would be maintained beyond the intervention period without continued yoga practice An additional limitation of the study was the significant gender imbalance between groups, which was not included as a covariate in the primary analyses due to the modest sample size. Although baseline outcome values were statistically controlled, the potential influence of gender on intervention responsiveness cannot be fully excluded and should be considered when interpreting the findings.

Furthermore, all outcome measures in the present study were clinical or questionnaire-based. Although these tools are well validated and widely used in geriatric research, the inclusion of objective biomechanical or instrumented balance assessments (e.g., force platform or wearable sensor–based measures) could have provided additional insight into postural control mechanisms and strengthened the conclusions.

## 6. Conclusions

This study demonstrated that a 12-week yoga program significantly improved balance, functional mobility, fear of falling, and mental health outcomes among older individuals residing in an old age home. These findings highlight yoga as a feasible, safe, and effective non-pharmacological approach to promote healthy aging and reduce fall-related risks. Integrating yoga into geriatric health promotion programs, particularly in institutional settings, may help older adults maintain independence, improve quality of life, and alleviate age-related functional decline. Future studies should explore larger, multi-site populations with longer follow-up to evaluate the long-term sustainability of these benefits and to establish standardized yoga protocols designed for older adults.

## Figures and Tables

**Table 1:** Demographic characteristics of the participants.

Variable	Yoga Group (n = 26)	Control Group (n = 24)	*p*-Value
Age (years) (Mean ± SD)	75.61 ± 7.10	77.12 ± 7.80	0.478
Gender n (%)			0.011
Male	7 (26.9)	16 (66.7)	
Female	19 (73.1)	8 (33.3)	
Marital status n (%)			0.349
Unmarried	1 (3.8)	1 (4.2)	
Married	16 (61.5)	19 (79.2)	
Widowed	9 (34.6)	4 (16.7)	
Education level n (%)			0.380
Illiterate	2 (7.7)	0	
Primary	1 (3.8)	0	
Highschool	9 (34.6)	6 (25)	
Graduation	10 (38.5)	14 (58.3)	
Postgraduation	4 (15.4)	4 (16.7)	
Living alone n (%)			0.153
Yes	15 (57.7)	9 (37.5)	
No	11 (42.3)	15 (62.5)	
Financial problem n (%)			0.589
Yes	2 (7.7)	4 (16.7)	
No	24 (92.3)	20 (83.3)	
Medication			0.318
Diabetes	9 (34.6)	11 (45.8)	
Hypertension	13 (50)	9 (37.5)	
Heart disease	2 (7.7)	4 (16.7)	
Pain killers	2 (7.7)	0	
Previous occupation			0.105
Service	18 (69.2)	18 (75)	
Business	1 (3.8)	4 (16.7)	
Homemaker	7 (26.9)	2 (8.3)	
Duration of stay at old age home (months) (Mean ± SD)	16.3 ± 11.06	13.4 ± 10.3	0.345

**Table 2:** Between-group comparisons with adjusted mean differences and 95% confidence intervals.

Measure	Group	Pre Mean (SD)	Post Mean (SD)	Within-Group Cohen’s d	Adjusted Mean Difference (95% CI)	*p*-Value
BBS	Yoga	43.48 (6.0)	49.88 (6.4)	1.04	6.20 (2.88 to 9.53)	<0.001
	Control	43.0 (12.2)	43.54 (7.1)	0.05		
FES	Yoga	41.35 (12.5)	32.81 (14.6)	0.62	−12.41 (−19.33 to −5.49)	0.001
	Control	41.75 (12.7)	45.38 (10.9)	0.30		
GAS	Yoga	5.46 (2.9)	3.38 (1.9)	0.84	−4.94 (−7.02 to −2.86)	<0.001
	Control	5.38 (4.5)	8.0 (4.3)	0.59		
GDS	Yoga	3.35 (1.4)	2.08 (1.8)	0.78	−2.32 (−3.59 to −1.06)	0.001
	Control	3.67 (3.2)	4.67 (3.9)	0.28		
TUG	Yoga	17.04 (8.3)	12.77 (4.6)	0.63	−9.09 (−11.69 to −6.50)	<0.001
	Control	20.58 (10.7)	24.54 (11.2)	0.36		

Notes: BBS—Berg balance scale, FES—Fall efficacy scale, GAS—Geriatric anxiety scale, GDS—Geriatric depression scale, TUG—Timed up and go test. Adjusted mean differences represent ANCOVA estimates controlling for baseline values. Positive values indicate improvement for BBS, while negative values indicate improvement for FES, GAS, GDS, and TUG.

## Data Availability

The individual data are available in the archives of the department and can be obtained from the corresponding author on request.

## Abbreviations

The following abbreviations are used in this manuscript:

BBS	Berg Balance Scale
TUG	Timed up and Go test
FES	Fall Efficacy Scale
GDS	Geriatric Depression Scale
GAS	Geriatric Anxiety Scale
ANCOVA	Analysis of Covariance
